# Engineered mice and B cell lines expressing broadly neutralizing antibodies and their unmutated precursors: tools for HIV vaccinology

**DOI:** 10.1186/1742-4690-9-S2-O41

**Published:** 2012-09-13

**Authors:** D Nemazee, C Doyle-Cooper, T Ota, AB Cooper, M Huber, E Falkowska, K Doores, L Hangartner, K Le, D Sok, J Jardine, J Lifson, X Wu, JR Mascola, P Poignard, JM Binley, BK Chakrabarti, WR Schief, RT Wyatt, DR Burton

**Affiliations:** 1 The Scripps Research Institute, La Jolla, CA, USA; 2 Frederick National Laboratory for Cancer Research, Frederick, MD, USA; 3 Vaccine Research Center NIAID, Bethesda, MD, USA; 4 Torrey Pines Institute, La Jolla, CA, USA; 5 IAVI Neutralizing Antibody Center, USA

## Background

Eliciting broadly neutralizing antibodies (bNAbs) to HIV Env through immunization has been problematic.

## Methods

To better understand the requirements for activation of B cells producing bNAbs, we generated a series of cell lines and transgenic mice expressing such antibodies or selected germline-reverted versions as B cell surface receptors. The bNAb cell lines included those that recognize the CD4 binding site (b12, VRC01, PGV04, PGV19, NIH45-46), the MPER of gp41 (4E10), and additional glycan-dependent sites on the trimer (PG9, PG16, PGT145, 2G12, PGT128, PGT135, PGT121). Different Env-containing antigens and virions were tested for the ability to stimulate bNAb cell lines.

Mouse strains expressing germline or mutated forms of 4E10 and b12 bNAb Ig genes were generated by gene targeting to the physiological loci. These “knock-in” mice were studied for their B cell development, and responses to HIV immunogens.

## Results

Many HIV Env antigen preparations, notably including infection-competent pseudovirions, were poorly recognized by high affinity bNAb-expressing cells, as measured by calcium flux assay. However, other antigen forms were highly stimulatory: in particular, soluble gp140 foldon trimers and a multimerized, scaffolded epitope protein.

4E10, but not b12 knock-in mice showed signs of abortive B cell development. b12 H mice had gp120-binding cells and responded well in vivo to gp140 trimers.

## Conclusion

Analysis of bNAb cell line activation suggested that HIV is difficult to recognize by B cells, probably because of the low density of surface proteins. Based on these results, soluble gp140 trimers or epitope scaffolds might offer more promise as vaccine candidates. In knock-in mice, primary 4E10 B cell precursors appeared to be negatively selected, whereas b12 B cells were normal and readily stimulated with gp140 trimers.

